# A Comparative Study on the Flocculation of Silica and China Clay with Chitosan and Synthetic Polyelectrolytes

**DOI:** 10.3390/md19020102

**Published:** 2021-02-10

**Authors:** Konstantin B. L. Borchert, Christine Steinbach, Simona Schwarz, Dana Schwarz

**Affiliations:** Leibniz-Institut Fuer Polymerforschung Dresden e.V., Hohe Straße 6, 01069 Dresden, Germany; borchert@ipfdd.de (K.B.L.B.); steinbach@ipfdd.de (C.S.); simsch@ipfdd.de (S.S.)

**Keywords:** chitosan, flocculation, solid/liquid separation, biopolymers

## Abstract

Flocculation is still one of the most important and efficient processes for water treatment. However, most industrial processes, such as in water treatment plants, still use huge amounts of synthetic polyelectrolytes for the flocculation process. Here we compare the flocculation of two different suspended particles, i.e., silica particles and china clay, with the biopolymer chitosan and two common strong synthetic polyelectrolytes. As a flocculant, chitosan featured a minimum uptake rate of 0.05 mg/g for silica and 1.8 mg/g for china clay. Polydiallyldimethylammonium chloride (PDADMAC) for comparison possessed a minimum uptake rate of 0.05 mg/g for silica and 2.2 mg/g for china clay. Chitosan as an environmentally friendly biopolymer competes with the synthetic polyelectrolytes and thus represents a beneficial economic alternative to synthetic flocculants.

## 1. Introduction

The sedimentation of dispersed solids is retarded by small particle sizes and electrostatic repulsion. To separate them from the aqueous phase, the dispersed particles must be combined into rapidly sedimenting aggregates by flocculation. Effective separation of solid particles in water downstream by water treatment plants is heavily reliant on flocculation with inorganic salts and synthetic polymers [1,2]. Flocculation as such is thereby reducing the amount of, e.g., inorganic particles and natural organic matter (NOM), by countering the surface charge of the suspended particles and therefore leading to aggregation of larger flocs and sedimentation of those. Because of aluminum and iron salts having different drawbacks, such as small floc size, high salt consumption, and a high sludge volume, a transition to the use of polymeric flocculants in industrial applications can be seen [1,2,3]. Polyelectrolytes are used in wastewater treatment as flocculants to separate dispersed solids. Commercially available flocculants differ in their chemical composition (i.e., charge type, charge content, and charge distribution) and steric structure (i.e., molar mass and molar mass distribution).

Although flocculation with synthetic polymers is a cost-effective, efficient, and facile water treatment method, it consumes huge amounts of synthetic polyelectrolytes whereby, e.g., toxic, unreacted monomers, or residual polyelectrolytes themselves can pose a risk to, e.g., aquatic life [4,5,6,7]. In order to achieve a turnaround of the economy to more sustainable and environmentally friendly practices, not only is the recovery of wastes or leftovers from, e.g., farm processes [8,9] important, but also more efficient methods for the treatment of polluted wastewaters to ensure cost-effectiveness and, furthermore, implementation as industrial standards [10].

One potential solution is the usage of biopolymers as flocculants, such as starch [11], chitosan [12], or other polysaccharides [13,14] with their biodegradable backbones, leading to environmental compatibility. Chitosan is a biopolymer, obtained from chitin by alkaline deacetylation of the *N*-acetyl-D-glucosamin groups of the chitin [12]. The properties of chitosan such as the solubility and positive charge at low pH values are greatly influenced by the molecular weight and the degree of deacetylation. Chitosan, either chemically modified or unmodified, is reported to be a viable adsorber material for the removal of heavy metal ions [15,16,17,18] and can also be applied for flocculation processes [19,20]. It was also reported to be an efficient flocculant for NOM itself [21] or together with humic acid for clay [22]. Divakaran and Pillai investigated the use of chitosan for the flocculation of kaolin and titanium dioxide and the influence of fulvic acid on the process [23].

As model system, often china clay (kaolin) or silica is tested. Different methods for the flocculation of these systems have been reported [14,22,23,24,25,26,27,28]. However, direct comparison of natural flocculants like chitosan and industrially widely applied synthetic polyelectrolytes, such as copolymers based on acrylamide (e.g., polyacrylamide with trimethylammonium chloride functionality, PAM-TAC) and polydiallyldimethylammonium chloride (PDADMAC), are rarely published.

To understand the process of flocculation, it is important to know that the stability of a suspension is influenced by multiple factors. The properties of the particles in the suspension, such as charge, size, and form, are important factors [29]. The other factors are the properties of the flocculant, such as zetapotential, charge density, chain length, and chain stiffness. The stability of the suspension can then be decreased by adsorbing polyelectrolytes of opposite charge to the particle’s surface. Depending on the conformation of the adsorbed polyelectrolyte and potential collisions of different particles, two fundamental mechanisms can lead to aggregation—the patch or the bridging mechanism. With often short-chained, flatly adsorbed polyelectrolyte chains that reduce the effective surface charge of the particle to zero, the patch mechanism is destabilizing suspensions by reducing the repulsive forces between particles. With a long-chained, more stiff, and space-taking conformation of the adsorbed polyelectrolytes, collision of different particles leads to their aggregation as the polyelectrolytes are adsorbed to both of them, therefore bridging them together [26,30]. Also, combinations of these two mechanisms are known as the synergistic use of polyelectrolyte complex flocculant systems [27,29].

In this study, we compare the suspension stability of spherical monodisperse silica particles with a diameter of 200 nm with the sheet silicate china clay with a diameter of around 300 nm. Those were employed as model systems, for sand and clays and silts, which are, among other contaminants, typical constituents in wastewater or natural waters. The suspension stability was influenced by the addition of polyelectrolytes yielding an aggregation and hence flocculation process of the particles. The influence of the properties of both particles as well as the type of polyelectrolyte used was investigated. Hence, we examined two commercially available synthetic strong cationic polyelectrolytes in comparison to the weak polyelectrolyte chitosan as a commercially available cationic biopolymer.

## 2. Results and Discussion

**Figure 1:**
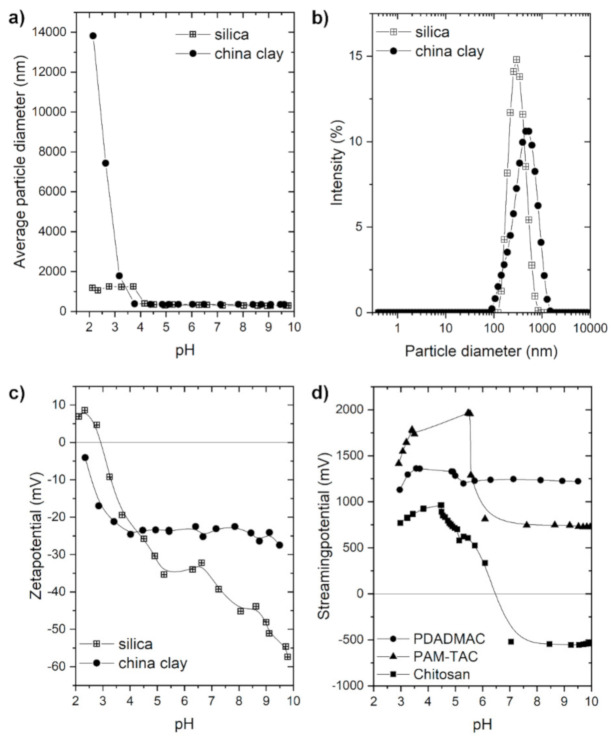
(**a**) Average particle diameter of silica and china clay in dependence of the pH, (**b**) particle size distribution of silica and china clay at pH = 5.6, (**c**) zetapotential of silica and china clay in dependence of the pH; with silica shown as crossed squares and china clay as filled circles. (**d**) Streaming potential of polydiallyldimethylammonium chloride (PDADMAC) (circles), polyacrylamide with trimethylammonium chloride functionality) PAM-TAC (triangles), and chitosan (squares) in dependence of the pH.

**Scheme 1:**
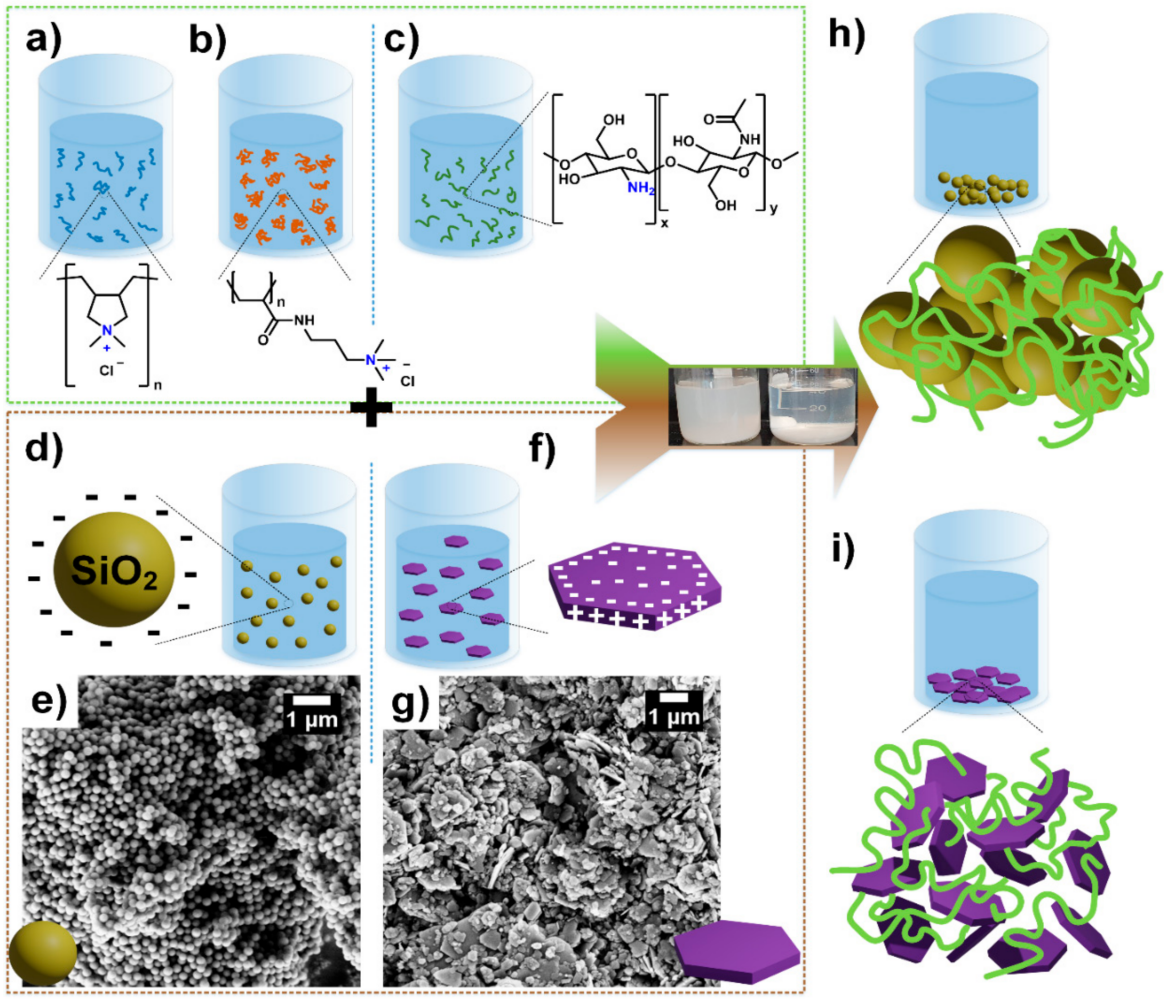
Schematic presentation of the flocculation process with the synthetic cationic polyelectrolytes (**a**) PDADMAC and (**b**) PAM-TAC (schematic partial structure of the cationic co-polymer) and the biopolymer (**c**) chitosan, which were used to investigate the flocculation process on (**d**) silica and (**f**) china clay. The graphic representation of the morphology and the surface charges of (**d**) silica and (**f**) china clay and the scanning electron microscopy (SEM) image of (**e**) silica and (**g**) china clay. The schematic flocculation process of (**h**) silica (yellow spheres) and (**i**) china clay (violet prism) with the cationic polyelectrolytes (displayed in light green color) is shown as well.

In this study, we compare the stability of the suspension containing spherical silica particles with the sheet silicate china clay. Hence, the pure silica particles and china clay were characterized by zetapotential, electron microscopy, particle size (see Figure 1 and Scheme 1), and nitrogen sorption measurements (see Figure S1 in the Supplementary Materials). The form of the two particles is different as the silica particles are monodisperse and spherical with a diameter of 200 nm. The china clay exhibits a sheet-like structure with a polydisperse particle size in the 300 nm range (see Figure 1b). Both inorganic particles featured a specific surface area of 16 m^2^/g calculated by multi-point Brunauer-Emmett-Teller (BET) method (Figure S1). Furthermore, both particles feature a mostly negatively charged surface over the pH range from 3 to 10 (see Figure 1c). The china clay did not reach the isoelectric point (IEP) at pH 2 but the zetapotential shows that the IEP of the china clay particles was close to pH 2. Thus, the china clay particles featured in the zetapotential measurement an overall negative charge over the whole examined pH range although the hexagonal platelets possess a negative charge on the faces and a positive charge on the edges. These charges are caused by the isomorphous substitution of Si and Al atoms by transition metal ions as impurities in the crystal structure of the mineral [31]. For comparison, the silica particles exhibited an IEP at pH 3 due to their homogeneously distributed silanol groups. Additionally, the particle size in dependence with the pH of the suspension was investigated (see Figure 1a). The particle sizes are constant at 200 nm for silica and 300 nm for china clay until pH 4 is reached. At lower pH values close to the IEP, the particles start to agglomerate due to the missing electrostatic interaction between them. For the silica particles, the particle size low pH 4 changes from 200 nm to a constant size of 1200 nm. For comparison, the china clay particles start to aggregate between pH 3 and 4 as well but the particle size increases with a decreasing pH value. At pH 2, the china clay particles reached a size of approximately 14 µm. Since both types of particles are barely charged at pH 2 (i.e., close to IEP), the difference in aggregated particle size needs to be explained by the particle form. The platelet-like form of the china clay yields in distinct larger aggregates since the stacking behavior and the surface interaction is a lot higher for platelets in comparison to spherical particles. The initial pH of the further investigated suspensions were 5.6 exhibiting a particle size of 200 nm for silica particles and 300 nm for china clay. The charge density of the particles in suspension at pH 5.6 was examined as well (see Table 1). The silica particles possessed a charge density of −0.0016 meq/g and the china clay particles of −0.0243 meq/g. Hence, the charge density of the silica particles was 15 times smaller than the charge density of the china clay suspension.

The stabilization of the suspension was examined by the addition of three different commercially available polyelectrolytes (PELs), i.e., the homopolymer polydiallyldimethylammonium chloride (PDADMAC), the cationic copolymers of acrylamide and acrylic acid derivatives (PAM-TAC), and the biopolymer chitosan. The two synthetic polyelectrolytes PDADMAC and PAM-TAC are common strong cationic synthetic polymers due to their quaternary ammonium groups. PDADMAC is a homopolymer featuring a cationic charge in each repeating unit (100 mol% charge fractions). The positively charged ammonium group is important for different liquid-solid separation processes including various different industry branches such as, e.g., water treatment applications, pretreatment of cellulose for the use in paper industry, and sludge dewatering [32,33,34]. Chitosan is a weak polyelectrolyte and a biopolymer. The structures of the three polymers are shown in Scheme 1. The here used PAM-TAC has the highest molar mass with approximately 6 mio. g/mol. PDADMAC for comparison, as the other strong PELs feature a relatively low molar mass of 37,000 g/mol. Chitosan as a biopolymer can feature various molar masses as well. For this investigation we chose a chitosan with a molar mass in the range of 150,000 to 300,000 g/mol. The chitosan solution commonly features a lower pH at around 4, as chitosan needs to be dissolved in an acid such as acetic acid or HCl. Table 1 shows the measured charge density of the three PELs at pH 5.6. For chitosan, the pH value had to be increased from the initial pH value of 4.1 to 5.6. Thus, the charge density of the three PELs decreased in the following order: PDADMAC (6.60 meq/g, pH independent), chitosan (5.41 meq/g), and PAM-TAC (2.59 meq/g). Furthermore, we measured the streaming potential for all three PELs in dependence of the pH. As expected, the two strong PELs, PDADMAC and PAM-TAC, showed a positive charge over the whole investigated pH range from pH 3 to pH 10 due to their quaternary amino group. The weak PEL chitosan featured the IEP at pH 6.5 and is therefore positively charged in the pH range below pH 6.5. Chitosan is not soluble at high pH values and can therefore only be applied as an efficient PEL for waste waters with a pH range between 3 and 6.5.

The stability of the suspension can be selectively controlled by the addition of PELs. The aim of this investigation is the solid/liquid separation and therefore the destabilization of the suspension. The solid/liquid separation by flocculation is an industrial process with high importance for, e.g., municipal sewage and paper making industry. Due to the different properties of the two types of particles, silica and china clay, the flocculation of these particles will differ.

**Figure 2:**
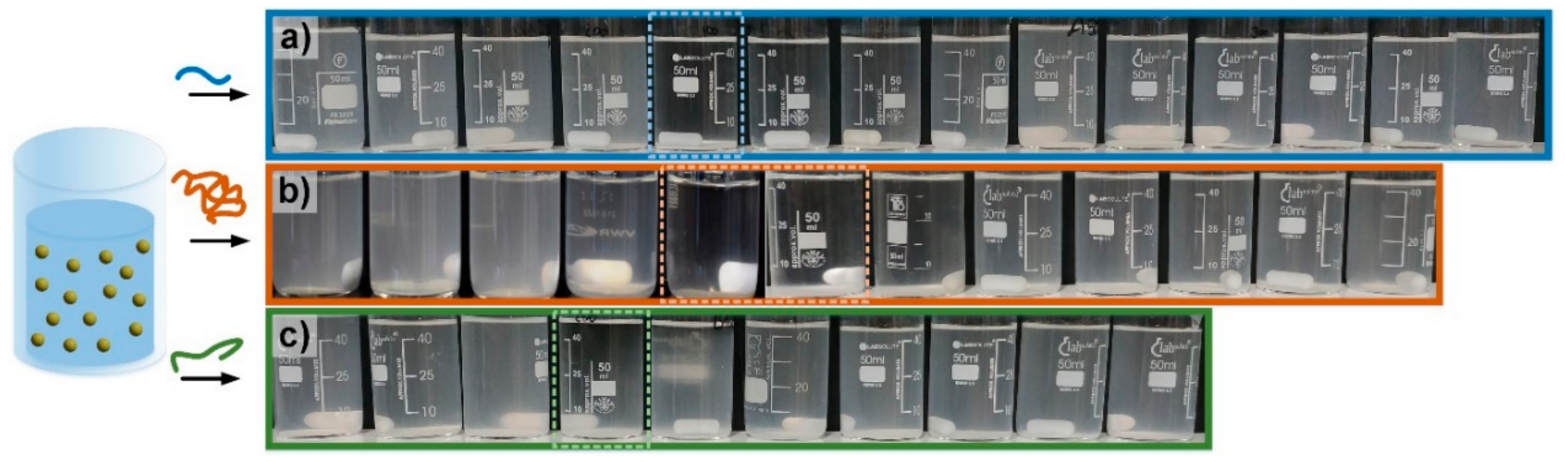
Flocculation images of silica (1 g/L) with (**a**) PDADMAC (0.1 g/L, blue) (**b**) PAM-TAC (1 g/L, red), and (**c**) chitosan (0.1 g/L, green). The box with the dotted line shows the flocculation window in the image. The amount of added polyelectrolyte (PEL) is increasing from *left-hand-side* to *right-hand-side* for (**a**) PDADMAC with *w_P_/w_S_*: 0.01 mg/g, 0.02 mg/g, 0.04 mg/g, 0.05 mg/g, 0.06 mg/g, 0.08 mg/g, 0.1 mg/g, 0.12 mg/g, 0.14 mg/g, 0.15 mg/g, 0.2 mg/g, 0.5 mg/g, 1.0 mg/g, 2.0 mg/g; (**b**) PAM-TAC with *w_P_/w_S_*: 0 mg/g, 0.08 mg/g, 0.12 mg/g, 0.16 mg/g, 0.2 mg/g, 0.3 mg/g, 0.4 mg/g, 0.5 mg/g, 1.0 mg/g, 2.0 mg/g, 3.0 mg/g, 4.0 mg/g, and (**c**) chitosan with *w_P_/w_S_*: 0 mg/g, 0.02 mg/g, 0.025 mg/g, 0.05 mg/g, 0.075 mg/g, 0.1 mg/g, 0.15 mg/g, 0.2 mg/g, 0.5 mg/g, 1.0 mg/g. The pH of the suspensions was 5.6. The image was taken shortly after stirring.

**Figure 3:**
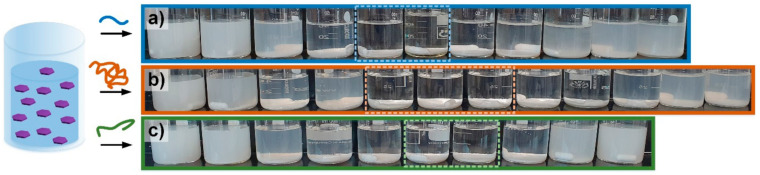
Flocculation images of china clay (1 g/L) with (**a**) PDADMAC (0.1 g/L, blue), (**b**) PAM-TAC (1 g/L, red), and (**c**) chitosan (0.1 g/L, green). The box with the dotted line shows the flocculation window in the image. The amount of added PEL is increasing from *left-hand-side* to *right-hand-side for* (**a**) PDADMAC with *w_P_/w_S_*: 1.0 mg/g, 2.0 mg/g, 2.2 mg/g, 2.5 mg/g, 2.8 mg/g, 3.5 mg/g, 3.7 mg/g, 4.0 mg/g, 4.5 mg/g, 5.0 mg/g, 6.0 mg/g; (**b**) PAM-TAC with *w_P_/w_S_*: 2.0 mg/g, 6.0 mg/g, 7.5 mg/g, 7.6 mg/g, 8.0 mg/g, 9.0 mg/g, 9.2 mg/g, 9.6 mg/g, 10.0 mg/g, 11.0 mg/g, 12.0 mg/g, 13.0 mg/g, and (**c**) chitosan with *w_P_/w_S_*: 0 mg/g, 1.0 mg/g, 1.6 mg/g, 1.8 mg/g, 2.0 mg/g, 2.2 mg/g, 2.4 mg/g, 4.0 mg/g, 4.7 mg/g, 6.0 mg/g. The pH of the suspensions was 5.6. The image was taken shortly after stirring.

**Figure 4:**
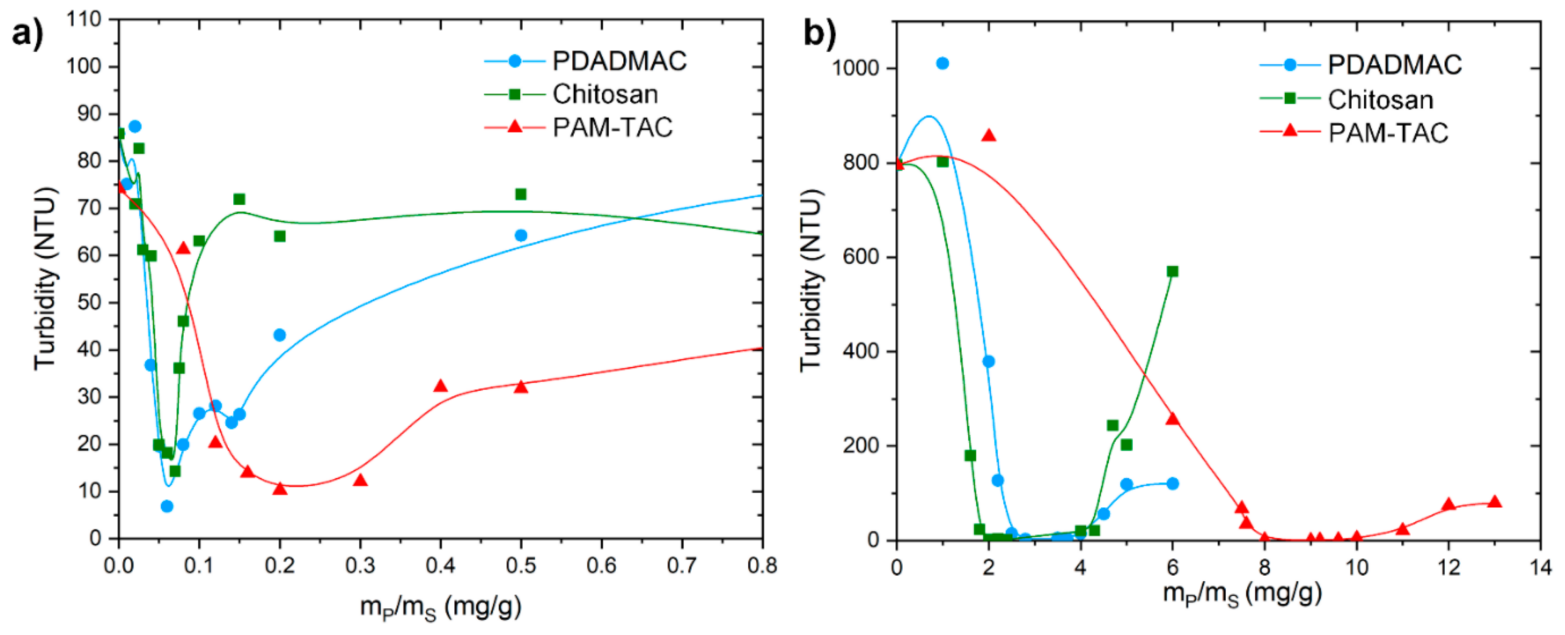
(**a**) Turbidity measurements of silica (1 g/L) with PDADMAC (0.1 g/L, blue spheres), PAM-TAC (1 g/L, red triangles), and chitosan (0.1 g/L, green squares). (**b**) Turbidity measurements of china clay (1 g/L) with PDADMAC (0.1 g/L, blue spheres), PAM-TAC (1 g/L, red triangles), and chitosan (0.1 g/L, green squares).

To compare the flocculation behavior of the two strong synthetic PELs and the weak biopolymer chitosan the inorganic particles silica and china clay were investigated as model system in a suspension. The turbidity of the silica and china clay suspensions after the addition of diverse amounts of the three PELs is displayed in Figure 2, Figure 3 and Figure 4. The images of the flocculation investigation with the silica suspension show light white and turbid suspensions as flocculation series with a flocculation window in the middle of the series where the solution becomes clear due to sedimentation of the mixture of silica-PEL. Before and after the flocculation window the silica solutions are stable aqueous suspensions because of electrostatic repulsion.

The images of the flocculation investigation with the three PEL and china clay are shown in Figure 3. The china clay suspension is clearly more turbid in comparison to the silica suspension although both solutions possess the same concentration. The difference in turbidity can be explained by the different particle size and surface charges (see Figure 1). The amount of PEL added in the displayed flocculation series for china clay is also relatively higher in comparison to the silica suspension. The flocculation windows for the three PELs can be clearly seen in the middle of each series.

The turbidity of the suspensions was estimated by turbidity measurements and is shown in dependency of the mass of the polymer *m_P_* and the mass of the substrate *m_S_* (i.e., *m_P_/m_S_*) (see Figure 4). Turbidity of the pure silica solution is around 80 NTU, tenfold lower than the turbidity for china clay at 800 NTU with the same concentration, which is in agreement with the images in Figure 2 and Figure 3. As expected, the flocculation window is broad for PAM-TAC, as this PEL features a low charge density and a high molar mass. The flocculation window is the sector with a very low turbidity. PAM-TAC features the broadest flocculation window in the *m_P_/m_S_* range of 0.15 mg/g to 0.3 mg/g for silica and in the *m_P_/m_S_* range of 7.5 mg/g to 11.5 mg/g for china clay. A broad flocculation window and therefore a broad region with a good flocculation can be beneficial for applications with a large fluctuation of parameters of the water, e.g., wastewater treatment plants and paper mills. Parameters of the water such as charge density of the suspended particles, amount of solid content, ionic strength, and pH can have a strong influence on the flocculation behavior. Hence, PAM-TAC would be a favorable PEL for this kind of requirement, as PAM-TAC features a large flocculation window in Figure 4. For industrial processes, costs are an important issue as well. Besides the broad flocculation window, PAM-TAC also has the highest *m_P_/m_S_* values yielding in a high consumption of PEL for the flocculation. Further investigations regarding the adsorption isotherm of PAM-TAC with kaolin (china clay) were reported elsewhere [35]. Hence, for processes with constant parameters the usage of PDADMAC and chitosan would be much more efficient.

The flocculation process is mainly based on the interaction of inorganic charged particles with positively charged PEL chains. Thus, one would expect that PEL with the highest charge density would lead to a low amount of PEL, which is necessary for an optimal solid/liquid separation. However, for both inorganic particles, silica and china clay, the biopolymer chitosan reached the lowest *m_P_/m_S_* values for a good flocculation. For the silica suspension, a flocculation with chitosan is reached at a *m_P_/m_S_* value of 0.05 mg/g. For comparison, PDADMAC reaches the flocculation window at *m_P_/m_S_* = 0.05 mg/g and for PAM-TAC at *m_P_/m_S_* = 0.15 mg/g. For the china clay suspension, chitosan reaches the flocculation window at a *m_P_/m_S_* value of 1.8 mg/g and PDADMAC at *m_P_/m_S_* = 2.2 mg/g and PAM-TAC at *m_P_/m_S_* = 7.8 mg/g. The *m_P_/m_S_* value for the china clay suspension is higher in comparison to the silica suspension due to the higher negative charge density of the china clay with −0.0243 meq/g. Silica features a charge density of −0.0016 meq/g. Hence, the amount of PEL added to reach the flocculation window for the china clay suspension is higher in comparison to the silica suspension.

**Figure 5:**
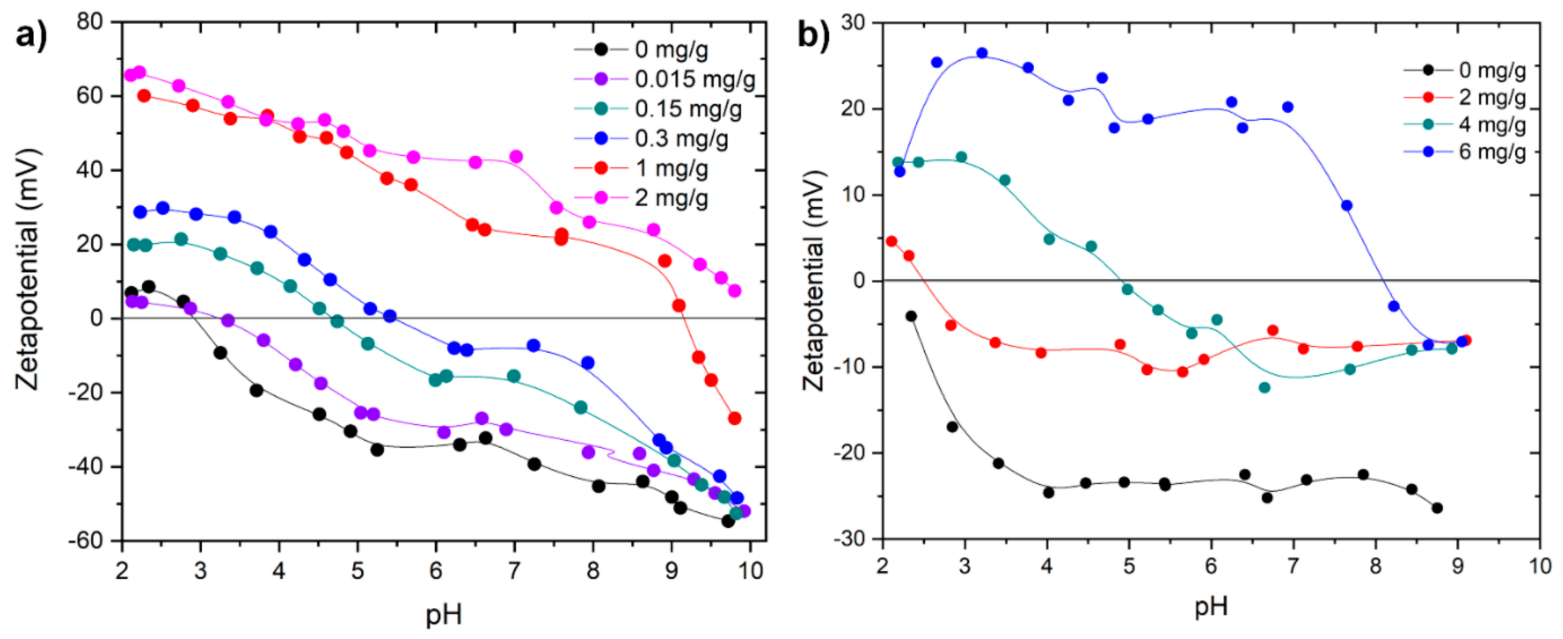
Zetapotential vs. pH measurements of (**a**) PDADMAC 0.1 g/L with silica 1 g/L and (**b**) PDADMAC 1 g/L with china clay 10 g/L.

Specific control of the stability behavior of suspensions is possible with PEL, as the charge of the particle surface changes with the adsorption of the oppositely charged PEL. Figure 5 shows the zetapotential vs. pH curves for the addition of different amounts of PDADMAC to a silica and china clay suspension, respectively. The cationic PDADMAC adsorbs on the surface of the negatively charged particles. If the amount of PELs exceeds the flocculation window, the positive charge on the particles’ surface increases due to the PEL yielding in a stable suspension again due to electrostatic forces. The IEP increases with increasing amount of PDADMAC in the suspension due to the positively charged PDADMAC. The IEP reached for the silica suspension with 1 mg/g PDADMAC at pH 9.5, the china clay suspension reached an IEP at pH 8 with 6 mg/g.

**Figure 6:**
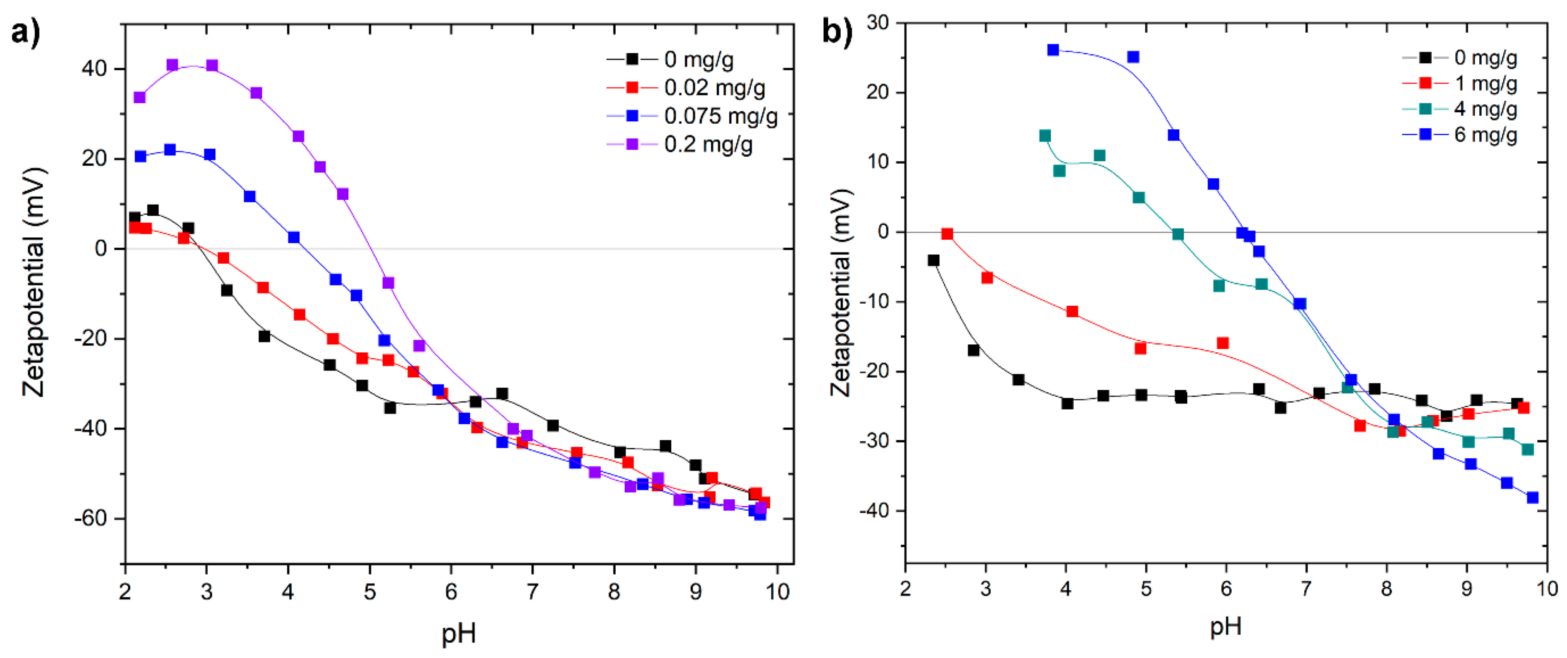
Zetapotential vs. pH measurements of (**a**) chitosan 0.1 g/L with silica 1 g/L and (**b**) chitosan 1 g/L with china clay 10 g/L.

Figure 6 shows the zetapotential vs. pH curves for silica and china clay suspensions treated with different amounts of chitosan. In general, both suspensions show an increasing IEP with an increasing amount of PEL in the suspension. With increasing amounts of chitosan, the positive charge on the particle surface increases due to the adsorption of chitosan. The flocculation optimum is reached at an addition of 0.05 mg/g for the silica suspensions. For clay suspensions the flocculation optimum is reached at an addition of 1.8 mg/g and the flocculation window is in the range of 1.8 mg/g to 4.2 mg/g (see Figure 4). Higher additions of 4.2 mg/g chitosan solution cause a restabilization yielding in an increase of the turbidity values again (see Figure 4). The zetapotential curve for 6 mg/g chitosan—a high excess of chitosan—in the china clay suspension featured a very similar curve progression to the pure chitosan. Chitosan possesses an IEP at pH 6.5 and becomes insoluble at higher pH values. Therefore, the zetapotential curve will always exhibit a negative charge at pH > 6.5 due to the insolubility of chitosan at high pH values.

**Figure 7:**
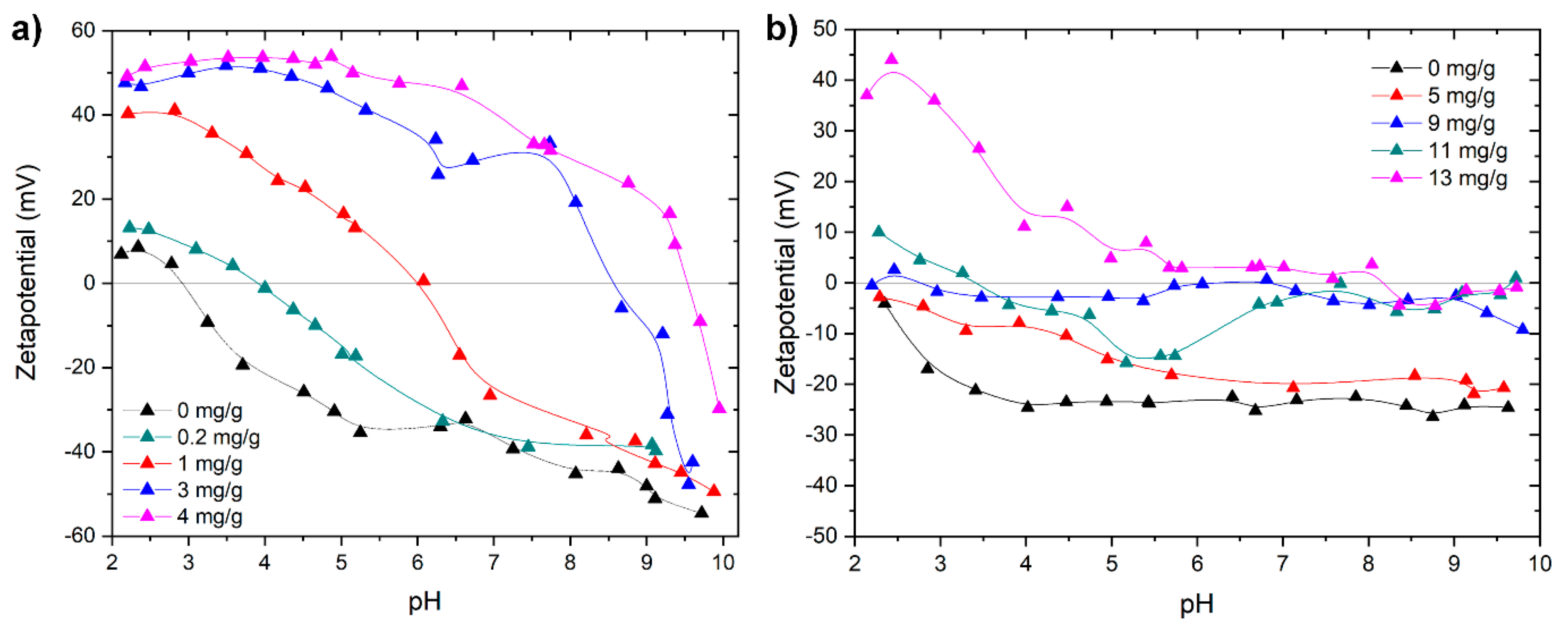
Zetapotential vs. pH measurements of PAM-TAC 1 g/L with (**a**) silica 1 g/L and (**b**) china clay 10 g/L.

Figure 7 shows the zetapotential vs. pH curves for silica and china clay suspensions treated with different amounts of PAM-TAC. When using PAM-TAC, the flocculation windows of silica suspension were in a range of 0.12–0.3 mg/g and for clay suspensions from 7.7–11 mg/g. The addition of 4 mg/g to the silica suspension shifts the IEP up to pH 9.5; 13 mg PAM-TAC per g clay results in a shift of the IEP to a pH of 8.3.

In general, it applies for all polymers studied that for polymer amounts larger than the respective flocculation range, there are sufficient positive charges on the particle surfaces that lead to a significant shift of the IEP with respect to the uncharged particle surface. Furthermore, the IEP is shifted toward higher pH values and the higher the positive charges are, the more restabilization occurs. Repulsive forces due to positive charges act now on the particle surface. In addition to the charge, the molar mass of the polymers is an important parameter. The lower the molar mass and the higher the charge, the higher the restabilization.

**Figure 8:**
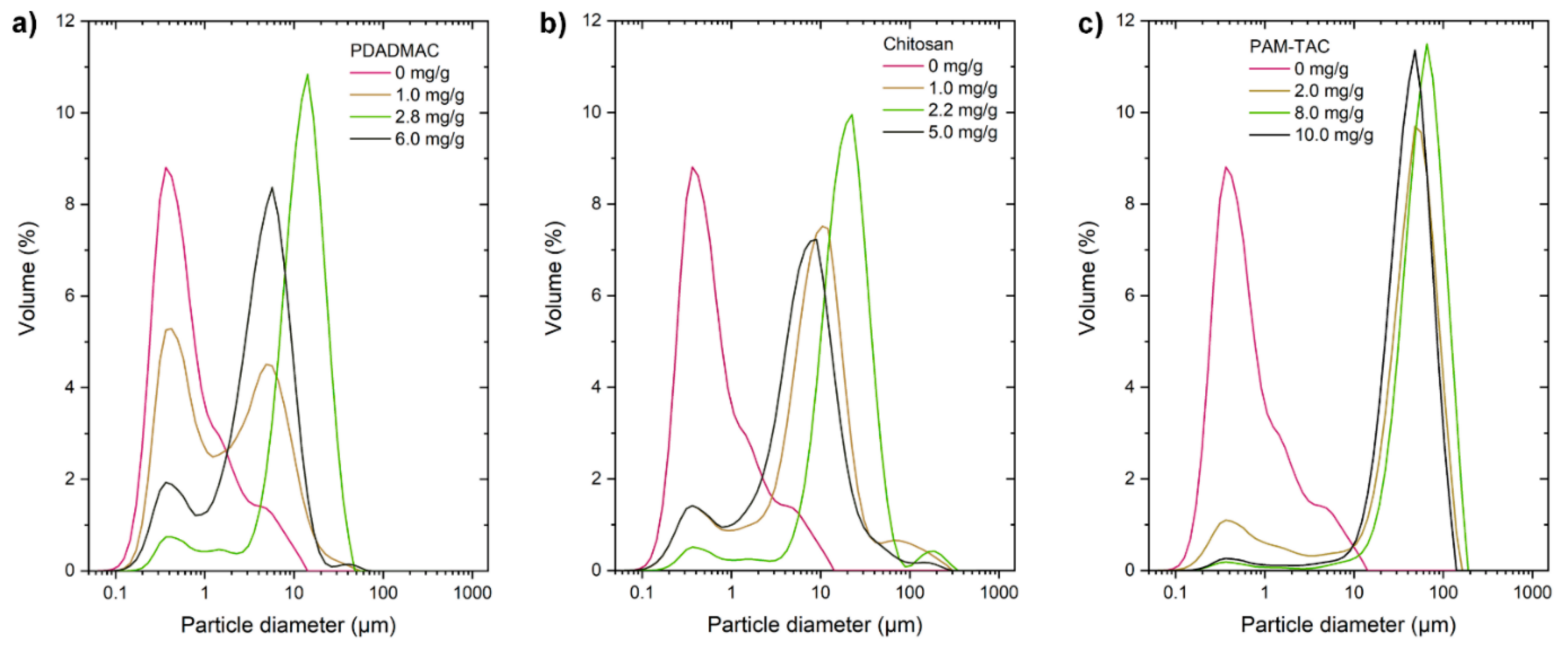
Particle size distribution of china clay (1 g/L) with (**a**) PDADMAC (0.1 g/L), (**b**) chitosan (0.1 g/L), (**c**) PAM-TAC (1 g/L).

The particle sizes as a function of polymer addition PDADMAC, chitosan, and PAM-TAC in clay suspensions are shown in Figure 8. The particle sizes were determined by laser diffraction at constant stirring of 2000 rpm. When PDADMAC and chitosan were used as flocculants, a fine fraction is still retained in addition to the large flocs. These flocculants do not give stable flocs under the effect of shear caused by shearing strain. PAM-TAC as flocculant gives shear stable and very large flocs up to 80 µm. This effect is potentially due to the high molecular weight of the PAM-TAC and the lower charge density, which allows shear stable flocculation via the bridging mechanism. In comparison, low molecular weight and high charge density like for PDADMAC and chitosan is more likely to flocculate via patch mechanism and yields in shear instable flocs [36,37,38].

High molecular weight, cationically modified polyacrylamides are used because only these ensure sufficiently high mechanical stability of flocculated slurries. Considerable issue with these acrylamide-based copolymers are the toxic residual monomer content and additives as well as the low biodegradability. The use of cationic polyacrylamides should be limited or avoided because of their high ecotoxicity. Product alternatives based on natural polymers derived from renewable raw materials such as starch, cellulose, or chitosan should be sought.

## 3. Materials and Methods

### 3.1. Materials

Chitosan in the form of flakes with a deacetylation degree of 85% (product name Ch85/200/A1) was purchased from the company BioLog Heppe^®^ GmbH (Landsberg, Germany). The product name Ch85/200/A1 refers to a deacetylation degree of 85%, a viscosity of 200 mPa∙s for 1 wt.% solution in 1 wt.% acetic acid, and an ash content of 1 wt.%, as provided by the manufacturer. China clay (CAS Number 1332-58-7) was purchased from Sigma-Aldrich (München, Germany). Powdered Silica with an identically particle size of 0.2 µm was purchased from Geltech, (Orlando, FL, USA). Polydiallyldimethylammonium chloride (PDADMAC, product name 40U50) with a molecular weight of 37.000 g/mol and a solid content of 42% was purchased from KATPOL-CHEMIE GmbH (Bitterfeld-Wolfen, Germany). PAM-TAC (product name: PRAESTOL^®^ 644BC) is a copolymer based on polyacrylamide (PAM) with cationic trimethylammonium chloride (TAC) groups and thus water soluble. It was purchased from Solenis (Schaffhausen, Germany). All chemicals were used as received.

For all experiments ultrapure water was used from MilliQ Advantage Q10 (Millipore, Darmstadt, Germany) with 3 ppm TOC and resistivity value of 18.2 MΩ·cm (25 °C).

### 3.2. Methods

#### 3.2.1. Zetapotential and Particle Size Measurement in Dependence of the pH

For the pure silica and china clay suspensions and after the addition of flocculants to the suspensions, the zetapotential vs. pH curves and particles sizes were measured with the device Zetasizer Nano ZS (Malvern Panalytical GmbH, Kassel, Germany). The samples were analyzed between pH 2 and 10 with a spacing between data points of 0.5 pH units. The pH was automatically adjusted by the addition/titration of HCl (0.1 M) and KOH (0.1 M). For each measurement, 15 mL of the sample was titrated from neutral pH (without adjustment) toward basic and acidic pH values.

#### 3.2.2. Particle Charge Detection (PCD) Measurements

Polyelectrolyte titrations were performed using a particle charge detector PCD-04 from the company BTG Instruments GmbH in Wessling, Germany. One milliliter of the polyelectrolyte solution (i.e., PDADMAC, PAM-TAC, and chitosan) and 9 mL of ultrapure water was titrated against a poly(sodium ethylenesulfonate) (PES) solution with a concentration of 0.001 M until the point of zero charge (isoelectric point, IEP) was reached. Ten milliliters of the suspensions (i.e., silica or china clay) were titrated against polydiallyldimethylammonium chloride (PDADMAC) solution with a concentration of 0.001 M until the IEP was reached. The pH values were determined during the measurement.

#### 3.2.3. Turbidity Measurements

After preparation of the flocculated sample (in experiments) the turbidity was analyzed with a Turbidimeter 2100AN IS (Hach Lange GmbH, Düsseldorf, Germany). The measurements were performed after 20 min of sedimentation of the sample.

#### 3.2.4. Particle Size Measurements

Particle size measurements of the flocs from the flocculation process were performed using a Mastersizer Microplus from the company Malvern. All samples were measured in ultrapure water with a constant stirring rate of 2000 rpm. The data was evaluated using the model “Polydisperse” and “OHD” (Particle RI: 1.5295, Abs.: 0.1000 and Dispersant RI: 1.3300).

#### 3.2.5. Nitrogen Sorption Measurements

Nitrogen sorption measurements were performed using the Autosorb iQ MP from Quantachrome Instruments (Boynton Beach, FL, USA). The samples were activated by degassing in vacuum (5 × 10^−10^ mbar) at 300 °C for 3 h. The nitrogen sorption measurements were performed at 77 K. For the calculation of the specific surface area, adsorption points of *p/p*_0_ in the range of 0.07 and 0.26 were used.

#### 3.2.6. Scanning Electron Microscopy (SEM)

Scanning electron microscopy was performed using the device SEM Ultra Plus from Carl Zeiss Microscopy GmbH, Jena, Germany. The samples were fixed with double-sided adhesive carbon tape on an aluminum pin sample tray and covered with carbon before the investigation starts. The measurements were carried out with an acceleration voltage of 6 keV at different magnifications.

### 3.3. Experiments

#### 3.3.1. Preparation of the Polyelectrolyte Solutions

Chitosan (1 g and 0.1 g in a 1 L volumetric flask each) were dissolved with 1 vol% glacial acetic acid in 1 L water and stirred for 24 h at rt, respectively. PDADMAC (1 g/L and 0.1 g/L) and PAM-TAC (1 g/L) solutions were prepared with water and stirred for at least 16 h at rt before use, respectively.

#### 3.3.2. Flocculation Experiments

One gram per liter silica and 10 g/L china clay were dispersed in water for 2 min by using a stick blender, respectively. The suspensions were immediately continued to use for the following flocculation experiments:

(a) The following flocculants were added separately to a 50 mL silica suspension:
  PAM-TAC, 1 g/L: 0–4 mg/g
  0 mg/g (pH 7.0), 0.2 mg/g (pH 6.8), 0.7 mg/g (pH 6.8), 1.0 mg/g (pH 6.9), 2.0 mg/g (pH 7.0), 3.0 mg/g (pH 6.9), 4.0 mg/g (pH 7.1).
  PDADMAC, 0.1 g/L: 0–2 mg/g
  0 mg/g (pH 7.0), 0.015 mg/g (pH 7.2), 0.05 mg/g (pH 7.0), 0.15 mg/g (pH 7.0), 0.30 mg/g (pH 7.2), 1.0 mg/g (pH 7.5), 2.0 mg/g (pH 7.8).
  Chitosan, 0.1 g/L: 0–0.2 mg/g
  0 mg/g (pH 7.0), 0.02 mg/g (pH 5.5), 0.025 mg/g (pH 5.4), 0.05 mg/g (pH 5.0), 0.075 mg/g (pH 4.9), 0.10 mg/g (pH 4.8), 0.20 mg/g (pH 4.4).
(b) The following flocculants were added separately to a 50 mL china clay-suspension:
  PAM-TAC, 1 g/L: 0–13 mg/g
  0 mg/g (pH 6.7), 5.0 mg/g (pH 7.3), 7.0 mg/g (pH 7.5), 9.0 mg/g (pH 7.4), 11.0 mg/g (pH 7.5), 13.0 mg/g (pH 7.5).
  PDADMAC, 1 g/L: 0–8 mg/g
  0 mg/g (pH 6.7), 2.0 mg/g (pH 7.7), 4.0 mg/g (pH 7.7), 5.0 mg/g (pH 7.5), 6.0 mg/g (pH 7.6), 8.0 mg/g (pH 7.6).
  Chitosan, 1 g/L: 0–6 mg/g
  0 mg/g (pH 6.6), 1.0 mg/g (pH 6.6), 2.0 mg/g (pH 5.9), 4.0 mg/g (pH 5.6), 6.0 mg/g (pH 4.6).

Subsequently, the suspensions were stirred with a magnetic stirrer for 15 min at 600 rpm and analyzed with the Zetasizer Nano ZS device (see Section 3.2.1). For the turbidity measurements, the samples sedimented after the stirring for 20 min before measurement [39].

## 4. Conclusions

PDAMAC, chitosan, and a cationic copolymer of polyacrylamide were used as flocculants for model suspensions, here silica and clay suspensions. The flocculants and the particles were characterized with respect to their charge density. Charge plays a major role in the solid/liquid separation process. For silica suspensions with a 15-fold lower negative charge compared to clay suspensions, lower amounts of PELs as flocculants were needed to set a flocculation window. Chitosan featured a very low polymer consumption and as a biopolymer is very suitable to be used as an alternative to synthetic polymers.

## Figures and Tables

**Table 1:** Molar mass of PDADMAC, chitosan, and PAM-TAC and charge density of the given polyelectrolyte solutions and suspensions of silica and china clay.

Sample	Molar Mass (g/mol)	Charge (meq/g)
PDADMAC	37,000	6.60
chitosan	150,000–300,000	5.41
PAM-TAC	ca. 6 mil.	2.59
silica	-	−0.0016
china clay	-	−0.0243

## Data Availability

Data is contained within the article or supplementary material.

## Supplementary Materials

The following are available online at https://www.mdpi.com/1660-3397/19/2/102/s1, Figure S1: N_2_ sorption isotherms measured at 77 K for china clay (full circles) and silica (crossed squares). Data points in the adsorption and desorption branch of the isotherms are indicated by black and gray coloration, respectively. The specific surface area of the china clay and silica was calculated to be 16 m^2^/g for both samples by multi-point BET method for 0.07 < *p/p*_0_ < 0.26.
