# Supplementary material for: A Comparative Study on the Flocculation of Silica and China Clay with Chitosan and Synthetic Polyelectrolytes
Source: Mar Drugs. 2021 Feb 10;19(2):102. doi: 10.3390/md19020102 (PMC7916584; doi:10.3390/md19020102)
Supplement: Supplementary file 1 [file marinedrugs-19-00102-s001.pdf]

ELECTRONIC SUPPLEMENTARY INFORMATION

for the manuscript

A Comparative Study on the Flocculation of Silica and China Clay with Chitosan and Synthetic Polyelectrolytes

Konstantin B. L. Borchert[1], Christine Steinbach[1], Simona Schwarz[1], Dana Schwarz[1,*]

[1] Leibniz-Institut fuer Polymerforschung Dresden e.V., Hohe Straße 6, 01069 Dresden, Germany; borchert@ipfdd.de (K.B.), steinbach@ipfdd.de (C.S.); simsch@ipfdd.de (S.S.); schwarz-dana@ipfdd.de (D.S.)

* Correspondence: schwarz-dana@ipfdd.de; Tel.: +49 351 46 58 542

Received: date; Accepted: date; Published: date

Abstract:

Flocculation is still one of the most important and extremely efficient processes for water treatment. However, most industrial process such as in water treatment plants still use huge amounts of synthetic polyelectrolytes for the flocculation process. Here we compare the flocculation of two different suspended particles, i.e. silica particles and china clay, with the biopolymer chitosan and two common strong synthetic polyelectrolytes. As a flocculant, chitosan featured a minimum uptake rate of 0.05 mg/g for silica and 1.8 mg/g for china clay. PDADMAC for comparison possessed a minimum uptake rate of 0.05 mg/g for silica and 2.2 mg/g for china clay. Chitosan as an environmentally friendly biopolymer competes with the synthetic polyelectrolytes and thus represents a beneficial economic alternative to synthetic flocculants.

1 Results

1.1 Nitrogen sorption measurements for silica and china clay

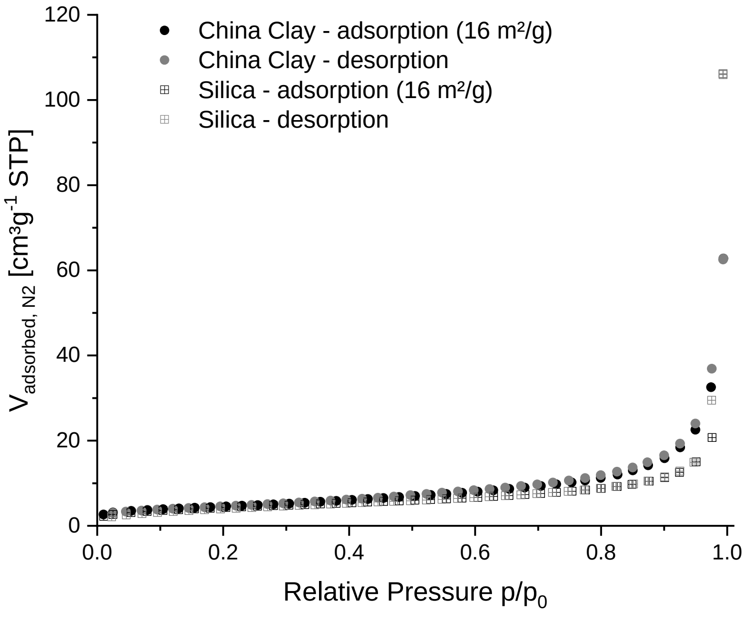

Figure S1: N_2 sorption isotherms measured at 77 K for china clay (full circles) and silica (crossed squares). Data points in the adsorption and desorption branch of the isotherms are indicated by black and gray coloration, respectively. The specific surface area of the china clay and silica was calculated to be 16 m^2/g for both samples by multi-point BET method for $0.07 < p/p_0 < 0.26$.
